# Evaluating Environmental Performance of PLA–Cellulose-Based Biocomposites: A Comprehensive Study on Biodegradability, Compostability, and Ecotoxicity

**DOI:** 10.3390/polym17233232

**Published:** 2025-12-04

**Authors:** Vera L. D. Costa, Pedro E. M. Videira, António de O. Mendes, Tomás Duarte, Bruno F. A. Valente, Paula Pinto, Alexandre Gaspar, Tânia Viana, Paulo T. Fiadeiro, Joana M. R. Curto, Maria Emília Amaral, Ana P. Costa, Joana C. Vieira

**Affiliations:** 1 Fiber Materials and Environmental Technologies Research Unit (FibEnTech-UBI), University da Beira Interior, R. Marquês D’Ávila e Bolama, 6201-001 Covilhã, Portugal; pedro.videira@ubi.pt (P.E.M.V.); fiadeiro@ubi.pt (P.T.F.); jmrc@ubi.pt (J.M.R.C.); mecca@ubi.pt (M.E.A.); anacosta@ubi.pt (A.P.C.); 2 Forest and Paper Research Institute (RAIZ), R. José Estevão, Eixo, 3800-783 Aveiro, Portugal; tomas.a.duarte@thenavigatorcompany.com (T.D.); paula.pinto@thenavigatorcompany.com (P.P.); alexandre.gaspar@thenavigatorcompany.com (A.G.); 3 GLNPlast S.A., E.N. 356-1, No. 24, Maceira, 2405-018 Leiria, Portugal; tania.viana@gln.pt

**Keywords:** biocomposites, biodegradability, compostability, ecotoxicity, PLA–cellulose-based material

## Abstract

Increasing concerns about environmental issues have recently intensified the search for sustainable alternatives to conventional plastics that minimize ecological impacts. This study evaluates the biodegradability, compostability, and ecotoxicity of a PLA-based biocomposite containing 30–40% micronized cellulose fibers. The material complied with the European limits for fluorine and heavy metals. Biodegradability was assessed through a respirometric test under thermophilic conditions, achieving 81% degradation in 155 days. Thermophilic compostability was evaluated by monitoring the disintegration of injected products made from the biocomposite pellets and cut into pieces with thicknesses of 1.0 mm and 2.1 mm, revealing that increased specific surface area prolongs composting time. Ecotoxicity was tested through seed germination and plant growth assays on barley, onion, sunflower, tomato, and wheat using the biocomposite mature compost mixed (25% and 50%) with a TÜV Austria certified soil. Results showed species-dependent effects: sunflower germination was enhanced, while other plants experienced slight growth delays. No severe phytotoxicity was observed, except for barley and wheat. Despite the proven biodegradability and compostability, the biocomposite product’s dimensions influence disintegration and decomposition rates. Furthermore, compost applications may have variable effects on plant development. These findings improved knowledge about sustainable materials performance, raising awareness about more responsible design, consumption, and disposal strategies.

## 1. Introduction

Growing concern about the environmental impacts associated with petroleum-derived synthetic polymers has driven research and development of sustainable and biodegradable alternatives. Composites formulated with conventional fossil-based polymers cannot be classified as biodegradable because they do not use bio-based polymer matrices. In contrast, materials that combine bio-based polymers with natural reinforcements are called biocomposites and have gained prominence in scientific research due to their smaller ecological footprint and potential to reduce environmental impact [1].

The growing demand for sustainable materials has led to the development of biocomposites based on polylactic acid (PLA) and cellulose, aiming to replace synthetic polymers for industrial and consumer applications [2]. Among the biopolymers that have emerged, PLA stands out for its renewable origin, biocompatibility, and mechanical performance comparable to those of conventional polymers such as polystyrene (PS) and polyethylene terephthalate (PET) [3,4,5]. PLA is a biodegradable aliphatic polyester with a preferentially hydrolytic degradation mechanism [1,5]. Its degradation occurs initially in the amorphous regions of the polymer chains but also influences the crystalline regions being affected by the percentage of crystallization of the material [6,7,8]. Although PLA is widely used in sectors such as packaging, textiles, biomedical, additive manufacturing (3D printing), and single-use products [1,5], its degradation time in soil is substantially longer than that of its biocomposites [9]. Although PLA is considered biodegradable, its degradation in natural environments can be slow and incomplete, particularly under low humidity and/or low temperature conditions [2]. The incorporation of cellulose, especially in the form of nanostructures such as microfibrillated cellulose (MFC), besides accelerating the degradation process, also plays an important role in improving mechanical performance and thermal properties [10]. On the other hand, the incorporation of natural fibers into the polymer matrix, namely cellulose fibers, has been an effective way to improve the performance of the biocomposites in the environment. These fibers, derived from renewable resources such as wood, enable the transport of water and enzymes into the material structure, promoting the fiber’s swelling and the consequent development of cracks in the biocomposite structure that lead to its accelerated degradation [6]. In addition, PLA drawbacks such as low thermal resistance and mechanical fragility have motivated the incorporation of natural reinforcements, such as cellulose, to produce biocomposites with improved performance. Cellulose, especially in its nanostructured form, offers significant advantages such as high mechanical strength, low density, and biodegradability [11]. The combination of PLA with nanocellulose has demonstrated substantial improvements in the thermal, mechanical, and barrier properties of the materials, making them promise for applications in packaging, civil construction, and biomedical devices [11].

Plant fibers are widely available, recyclable, non-toxic, biodegradable, non-abrasive, and inexpensive, in addition to requiring less energy to produce compared to synthetic fibers [9,12]. The main constituents of all plant fibers are cellulose polymer chains, hemicelluloses and lignin, and the proportions of each depend on the plant species. Cellulose is a semicrystalline polysaccharide composed of D-glycosidic bonds which contains a large number of hydroxyl groups (three in each repeating unit), providing it with its characteristic hydrophilic properties [12].

Despite advances in the formulation and physical–chemical characterization of PLA–cellulose biocomposites, there remains a gap in assessing their environmental performance and safety, particularly regarding biodegradability, compostability/disintegration, and ecotoxicity. These parameters are essential for validating the sustainability of materials throughout their life cycle and ensuring that their degradation does not pose ecological risks. Compostability is assessed by disintegration and biodegradation, which are two complementary processes. Compostability assessment is defined and standardized by EN 13432 [13] and EN 14995 [14] and requires verification of four criteria:

1. Chemical characterization: A minimum of volatile solids content of 50% (this parameter is used to estimate the amount of organic material susceptible of being degraded), and limit values for heavy metals (values listed in Annex A of EN 13432 [13]);
2. Biodegradability: >90% of the organic material is converted to CO_2_ in 6 months of thermophilic testing (respirometric method according to ISO 14855-1 [15], and gravimetric CO_2_ measurement method according to ISO 14855-2 [16]);
3. Compostability/Disintegration: <10% residue after sieving through a 2 mm sieve, in 84 days of thermophilic composting (laboratory test according to ISO 20200 [17], and pilot test according to ISO 16929 [18]);
4. Compost quality: The final quality of the mature compost should not negatively affect plant growth—plant ecotoxicity test (OECD 208 [19]).

Biodegradability refers to a material’s ability to be decomposed by microorganisms under natural environmental conditions. Studies show that PLA–cellulose biocomposites exhibit a higher degradation rate in soil compared to pure PLA due to the presence of cellulose, which favors water retention and microbial colonization [2]. Degradation is evidenced by mass loss, morphological changes that can be observed by scanning electron microscopy (SEM), and ester bond cleavage that can be detected by Fourier Transform Infrared Spectroscopy (FTIR). The products obtained through the degradation process can be water, carbon dioxide, methane, biomass, and humic matter, depending on whether biodegradation occurred in biotic or abiotic conditions [8,20].

Compostability (achieved by complete disintegration of the material) is a specific form of degradation that occurs under controlled conditions of temperature, humidity, and in the presence of oxygen, similar to those of industrial composting procedures. PLA–cellulose biocomposites usually decompose well in industrial composting environments (i.e., under thermophilic conditions, since the glass transition temperature of the PLA has to be achieved for an effective degradation) [21], initially degrading the amorphous phase of the polymer and promoting an increase in the material’s residual crystallinity, indicating an efficient disintegration [2]. Concomitantly, the cellulose incorporated into the biopolymer matrix acts as a structuring agent, promoting aeration and overall facilitating the progression of the process.

While biodegradability and compostability are desirable attributes of a given product destined for disposal, it is essential to evaluate the ecotoxicological effects of the compost that remain after its disintegration/degradation because it may contain toxic additives that are potentially harmful to seed germination, plant growth, and overall soil health. Ecotoxicological testing of mature compost applied to plants such as *Lactuca sativa* (lettuce) and *Zea mays* (corn) has been extensively performed in order to determine the phytotoxicity of the degradation products present in the compost. Preliminary results from these tests indicate that PLA–cellulose biocomposites generally have no adverse effects on plant growth, reinforcing their potential use as environmentally safe materials [11]. Furthermore, it is essential to implement social strategies that educate consumers and businesses in the most appropriate way to manage bio-based products. Such strategies help prevent improper waste disposal in environments where biodegradation can be limited and fight greenwashing [20].

Regarding end-of-life of PLA-based products, because these materials are only compostable under specific controlled conditions (temperature > 58 °C, humidity and microbial activity), industrial composting emerges as the preferred route, requiring a minimum conversion of 90% to CO_2_ within 180 days and the absence of toxic residues due to environmental safety concerns [22]. Alternatively, mechanical or chemical recycling methods are valid paths but still face major technical challenges such as maintaining the material’s properties and economic viability [23]. Although PLA is compostable and biodegradable, the integration of cellulose can improve mechanical and thermal properties (expanding the applicability of this material) while concurrently accelerating its physical disintegration and favoring biomass formation upon composting [24,25]. Therefore, the use of lignocellulosic waste as reinforcement in PLA-biocomposites can emerge as a strategic way of contributing to the circular economy, mitigating environmental impacts and adding value to agricultural byproducts [26].

In this context, this work aims to address the lack of information on the environmental assessment of PLA–cellulose biocomposites by analyzing their behavior under different geometries when subjected to the same degradation/disintegration conditions and the phytotoxic effects resulting from the application of the obtained compost. The methodology used included laboratory biodegradation tests in soil by respirometry and disintegration in a simulated industrial environment (thermophilic incubation), as well as ecotoxicological tests with plants. The results obtained from this research can help to understand the potential environmental impacts resulting from the disposal or application of composts derived from these biocomposites and help to develop guidelines for their sustainable use and safe application in the environment.

## 2. Materials and Methods

### 2.1. Materials

The objective of this investigation was to study the biodegradability, compostability, and ecotoxicity of a PLA-based biocomposite, evaluating its potential as a sustainable and safe alternative material. Biodegradability determination in a soil medium was performed using appropriate commercial soils as a degradation bed under thermophilic incubation. The samples consisted of PLA pellets (6 × 2 × 2 mm) with a content of micronized cellulose fibers ranging from 30 to 40%, henceforth designated by BCP. The micronized cellulose fibers were obtained from bleached eucalyptus kraft pulp (BEKP), supplied by The Navigator Company (Aveiro, Portugal), using specialized milling equipment. The PLA used was Ingeo 3251D grade (NatureWorks, Minneapolis, MN, USA), with a melt flow index (MFI) of 80 g/10 min at 210 °C under a load of 2.16 kg. From these pellets, two products with diverse thicknesses (1.0 mm—IP1, and 2.1 mm—IP2) were produced by injection-molding and 2.5 cm square pieces cut from the products were subsequently buried under Synthetic Solid Waste (SSW) in order to determine the time required for complete disintegration under thermophilic composting conditions. The mature compost derived from the pellet’s composting procedure was then mixed at 25% and 50% weight ratios with a TÜV Austria-certified soil and applied in ecotoxicological assays focused on seed germination and plant growth, employing five different plant species, namely barley (*Hordeum vulgare*), onion (*Allium cepa*), sunflower (*Helianthus annuus*), tomato (*Solanum lycopersicum*), and wheat (*Triticum aestivum*).

### 2.2. Methods

BCP were compounded on a pilot-scale extrusion line equipped with a co-rotating twin-screw extruder (ZSE 35 iMAXX, L/D = 48, screw diameter 35 mm; Leistritz Extrusion Technology, Nuremberg, Germany). The system included a double intermeshing spindle, three independent feeders, and an underwater pelletizer. Processing parameters comprised a screw speed of 155 rpm, vacuum degassing at 100 mbar, and a temperature profile ranging from 160 °C to 180 °C along the barrel.

Injection-molded specimens were produced using two Engel Victory machines with different clamping capacities: an 80-ton unit for IP1 and 300-ton unit for IP2 (Engel, Schwertberg, Austria). Both machines operated under the same barrel temperature profile (180-175-170-165-160) with a mold temperature of 20 °C and an injection speed of 115 mm/s. Prior to molding, pellets were dried at 80 °C for 6 h in a dehumidifier to prevent moisture-related defects.

For the initial characterization, the samples were analyzed in terms of their volatile solids content and the quantification of heavy metals to verify compliance with the limit values established by European standards NP EN 13432 [13], EN 14995 [14], and ISO 18602 [27]. In agreement with ISO 18602 [27], fluoride concentrations were determined through ion chromatography, while the heavy metals (As, Co, Cr, Cu, Mo, Ni, Se, Zn, Cd, Hg, Pb) were quantified using inductively coupled plasma atomic emission spectroscopy (ICP-OES). The three samples (Biocomposite Pellets—BCP, Injected Part 1—IP1 and Injected Part 2—IP2) were also analyzed by Attenuated Total Reflection Fourier Transform Infrared Spectroscopy (FTIR-ATR) and X-ray Diffraction (X-RD) in order to complement the characterization of the materials. The FTIR spectrum of each material provides a unique profile that can be used for identification and comparison of different materials, like a “fingerprint”. To perform this test, 64 scans were selected, with a resolution of 4 cm^−1^ in the FTIR-ATR spectrometer Thermo Scientific Nicolet^TM^ iS^TM^10 (Thermo Fisher Scientific™, Waltham, MA, USA). On the other hand, from the X-RD analysis, we were able to obtain information about the crystallinity of the material. For this test, a CuKα radiation (λ = 1.54 Å), a voltage of 40 kV, a current of 30 mA, a scan of 5 to 40° and a step of 0.05° were used and performed in a Rigaku diffractometer, model DMAX III/C (Rigaku Holdings Corporation, Tokyo, Japan).

In order to follow the requirements of EN 14995 [14], aerobic composting tests were conducted to evaluate disintegration by composting, according to the ISO 20200 [17] methodology, which simulates composting under controlled laboratory conditions, using SSW as a composting bed. According to ISO 20200 [17], the SSW mixture prepared as a composting bed for simulated industrial composting is formulated to maintain a C:N ratio between 20:1 and 40:1 and appropriate moisture content, ensuring a suitable aerobic environment. The typical composition includes sawdust as the main source of carbon and structure, rabbit feed, mature compost to provide additional microbial inoculum, corn starch, sucrose, corn oil, and urea as a nitrogen source. This mixture is maintained under controlled conditions, generally at a temperature of around 58 °C, with adjusted humidity and aeration, to evaluate the disintegration of plastics or biocomposites. Each composting assay was performed in triplicate. A mature compost was thus produced from the sample, and its complete disintegration was evaluated under thermophilic conditions, in accordance with ISO 20200 [17]. Parameters examined at the end of the composting process included the compost’s visual appearance, smell, pH level, carbon-to-nitrogen (C:N) ratio, dry matter content, volatile solids content, and degree of disintegration of the sample in each corresponding compost (the latter was attained through sieving the compost and looking for any undisintegrated pieces).

To assess potentially hazardous effects on living organisms, ecotoxicity tests were performed applying the compost previously produced. Two types of tests were undertaken: a 21-day test to evaluate phytotoxic effects on plants’ growth, following OECD 208 [19] and NP EN 13432 guidelines [13], and another adapted from ISO 18763 to assess phytotoxicity during seed germination and initial root development over a period of 72 h [28]. The growth tests were carried out over 21 days, using two compost–soil mixtures (50:50 wt.% and 25:75 wt.%) tested in triplicate for each condition, alongside a control group consisting of 100% soil. Five seeds were planted per pot (making 15 seeds of each species for each of the substrate compositions), and all pots were placed on trays and irrigated with distilled water as needed. Plant height was measured periodically, and photographic documentation was collected throughout the experiment. Growth rates were calculated relative to the control group. The experiments were conducted in climate-controlled cultivation tents maintained at temperatures between 12 °C and 32 °C, with a relative humidity of 70 ± 25% RH and a photoperiod of 16 h of light and 8 h of darkness. At the conclusion of the tests, plants were inspected for abnormalities in stem and leaf morphology.

For the germination assay, a leachate was prepared by mixing compost obtained from the previously performed aerobic composting tests with distilled water in a weight proportion of 1:10 at room temperature under agitation for two hours, followed by filtration. Germination tests were performed using an adaptation from ISO 18763 [28] procedure, where Petri dishes were lined with germination-grade filter paper in triplicate, with ten seeds of each species per dish (making a total of 30 germinated seeds of each species in both control and leachate). Barley, wheat, and sunflower were selected for this germination test considering that these three species have similar germination rates. Each test dish was provided with 5 mL of leachate, while control dishes were irrigated with the same amount of distilled water. Daily photographs were taken, and after 72 h, root lengths were measured. Mean root lengths and germination indexes were calculated relative to the control groups for each species.

Biodegradability assays were conducted following the ISO 14855-1 [15], which evaluates the final aerobic biodegradation of plastic materials in a solid medium using a closed respirometer system (Echo Instruments, Zeče, Slovenske Konjice, Slovenia). Samples subjected to biodegradability assays were analyzed beforehand in terms of Total Organic Carbon content (TOC) according to the standards of ISO 10694 [29] and ISO 10693 [30]. The respirometric method involves monitoring CO_2_ production under controlled composting conditions (58 ± 2 °C, in the dark) for a maximum period of six months. The percentage of biodegradation is calculated based on the cumulative CO_2_ produced relative to the theoretical maximum, determined from the **TOC content** of the test material against the CO_2_ production of blank reactors containing the same amount of soil but no test sample. Each test required a minimum of 50 g of dry solids containing at least 20 g of TOC per reaction vessel. The test material was used in its original pellet form (maximum surface area ~2 × 2 cm). The respirometric assay was performed in triplicate, in which a total of nine reaction vessels were prepared: three blanks (only soil), three vessels with a reference material (microcrystalline cellulose—MCC, 20 µm), and three vessels with the test sample. The validity of the test was confirmed through three checkpoints: (1) CO_2_ production in blanks Between 50 and 150 mg/g of volatile solids after 10 days; (2) >70% biodegradation of the reference material after 45 days; and (3) <20% variation in biodegradation among vessels containing the reference material at the end of the test. Weekly maintenance was performed on each vessel to mix and aerate the mixture and to adjust moisture if needed. The biodegradability test is ended after an extent of six months or when a calculated biodegradation plateau is reached for each sample.

## 3. Results

The first stage of this study consisted of determining the volatile solids content, as well as analyzing the presence of heavy metals and other toxic substances, in order to meet the first requirement established for assessing compostability. The volatile solids content obtained for the three sample types is listed in Table 1.

The validity of the tests was confirmed by the whitish coloration of the ashes, characteristic of slow incineration throughout the heating ramp, and the values meet the minimum of 50% required by European standards NP EN 13432 [13] and EN 14995 [14] for further composting and biodegradation testing. Table 2 presents the results obtained in the analysis of heavy metals and other toxic substances for the cellulose-based samples under study.

The analysis of Table 2 confirms that the samples meet the limits imposed by the European standards NP EN 13432 [13] and EN 14995 [14] for heavy metals and other toxic substances.

**Figure 1:**
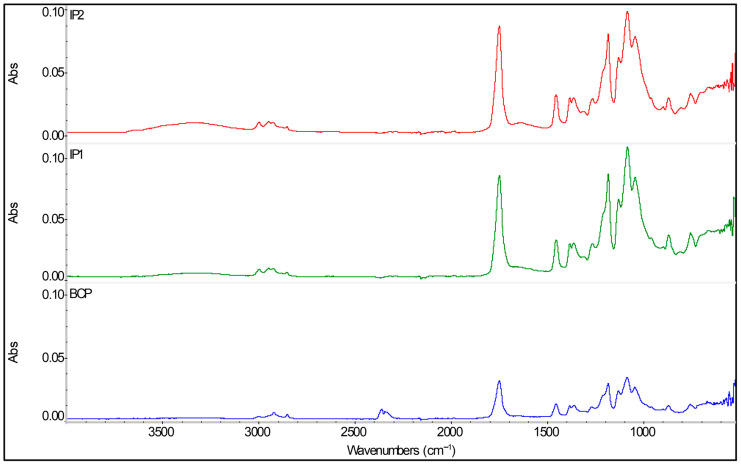
Graph obtained by FTIR-ATR of the 2 injected parts (IP1 and IP2) and the biocomposite pellets (BCP).

**Figure 2:**
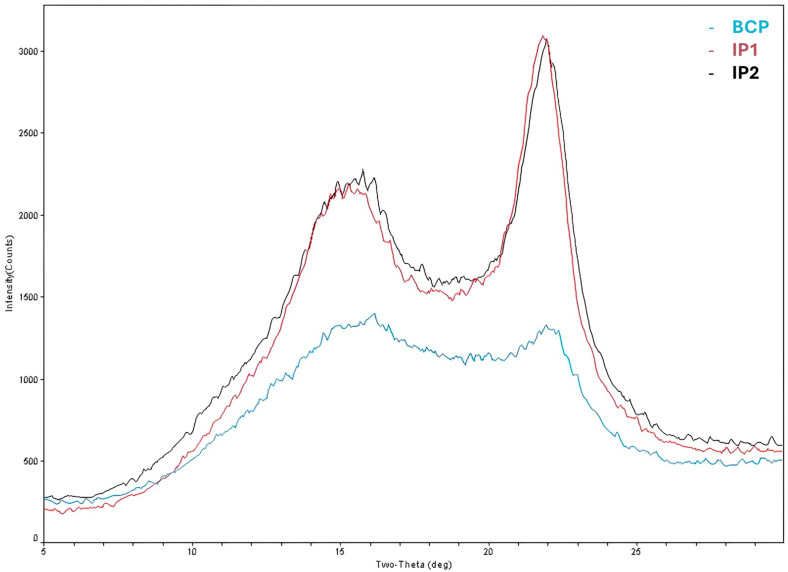
Graph obtained by X-RD of the 2 injected parts (IP1—red line and IP2—black line) and the biocomposite pellets (BCP—blue line).

Figure 1 shows the graphs for comparing the FTIR-ATR spectra of the two different samples produced by the injection molding process (IP1 and IP2) and from the biocomposite pellets (BCP). The X-RD graphs obtained from these samples (IP1, IP2, and BCP) are shown in Figure 2. The determination of the crystallinity index of the samples was carried out using the Segal method (the most widely used by the scientific community) [31]. The calculations for determining the crystallinity index (CrI) were performed according to Equation (1), Segal’s equation [31,32]:

CrI (Segal) = [(I_002_ − I_am_)/I_002_] × 100 (1)

where I_002_ is the maximum intensity of the crystalline peak and I_am_ is minimum intensity in the amorphous region. Table 3 presents the calculated CrI values for the three samples, using the Segal method. Because the Segal method is empirical and sensitive to peak overlap and background definition, these CrI values should be interpreted as relative indicators of increased crystallinity within this set of samples, rather than as absolute measures of crystallinity.

**Figure 3:**
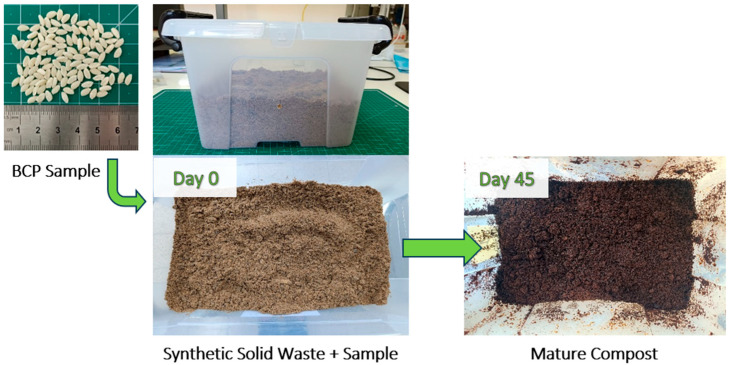
Appearance evolution of the SSW (sample BCP) from the beginning of the disintegration test to the end, obtaining a mature compost.

The next stage of this work was to compost the samples under thermophilic conditions (58 ± 2 °C) until 100% disintegration in accordance with the ISO 20200 standard [17]. This aim was accomplished after 45 days for the BCP, 106 days for IP1, and 141 days for IP2. Figure 3 illustrates the appearance evolution of the SSW from the beginning of the test (day 0) to a mature compost obtained at the end of BCP thermophilic composting (day 45).

**Figure 4:**
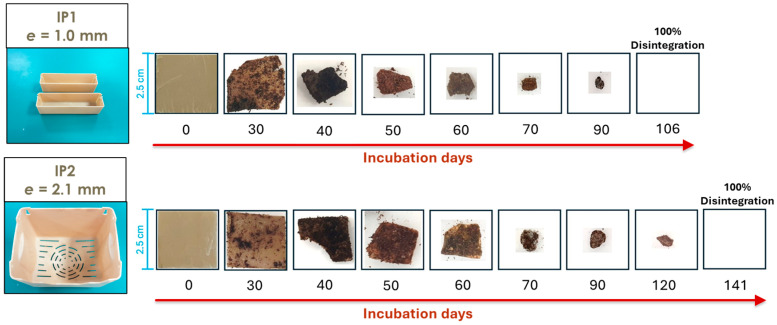
Material appearance evolution (samples IP1 and IP2) throughout the disintegration test.

Figure 4 shows the disintegration progression during the thermophilic incubation for the IP1 and IP2. Table 4 summarizes the results obtained for each disintegration test.

**Figure 5:**
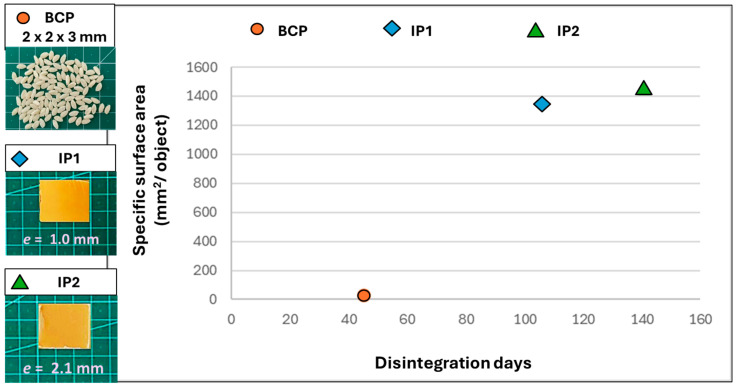
Correlation between the samples’ specific surface area and the days until complete disintegration during the composting tests under thermophilic incubation.

The plot shown in Figure 5 aims to illustrate how the thermophilic disintegration time evolved with the increase in the specific surface area calculated for each sample.

This work proceeded with the phytotoxicity assessment, using the obtained mature compost of the BCP sample. Regarding the germination test, after 72 h the average root lengths were determined, and the germination index (*GI*) was assessed against a control group (germinated in distilled water), through Equation (2) [33]:

*GI*(%) = [*RG*(%) × *RRL*(%)]/100, (2)

where *RG* is the relative germination in percentage and *RRL* is the relative root length in percentage. These two parameters can be calculated using the following Equations (3) and (4), respectively:

*RG*(%) = (N_GS,*T*_/N_GS,C_) × 100, (3)

*RRL*(%) = [L_R,*T*_ (mm)/L_R,C_ (mm)] × 100, (4)

where N_GS,*T*_ is the arithmetic average of the number of germinated seeds from the test triplicate, N_GS,C_ is the arithmetic average of the number of germinated seeds from the control, L_R,*T*_ is the mean root length of the test triplicate in millimeters, and L_R,C_ is the mean root length of the control in millimeters.

In order to ensure a reliable assessment of the compost’s effect on seed germination, a GI over 100% was interpreted as indicative of a germination fertilizing effect. Conversely, GIs within the ranges of 80–100%, 60–80%, and below 60% were classified as slightly, moderately, and severely phytotoxic, respectively.

Table 5 organizes the results attained for the 72 h seed germination tests, which were the subject of the first ecotoxicity evaluation of the mature compost obtained for the BCP sample.

**Figure 6:**
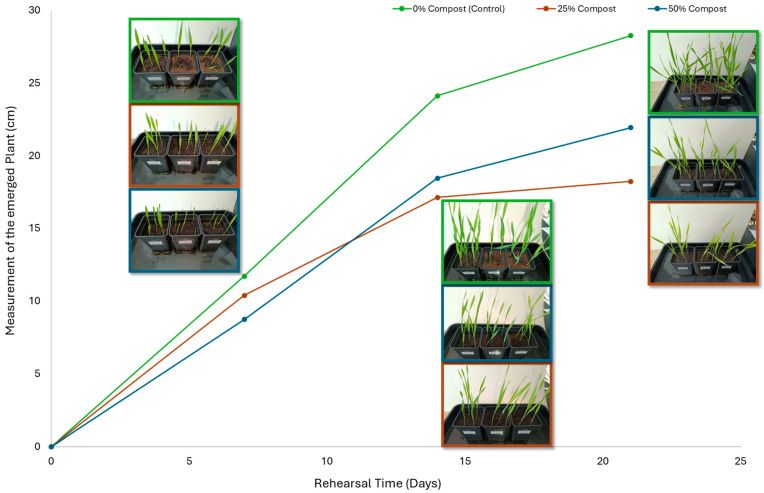
Barley growth evolution during the plant growth test.

**Figure 7:**
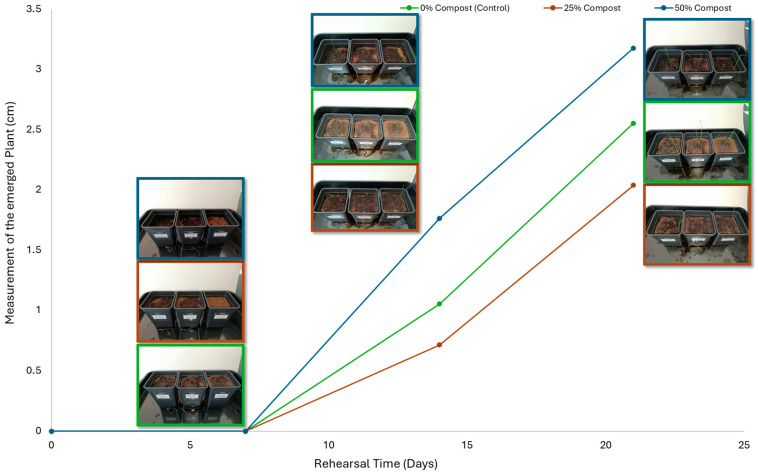
Onion growth evolution during the plant growth test.

**Figure 8:**
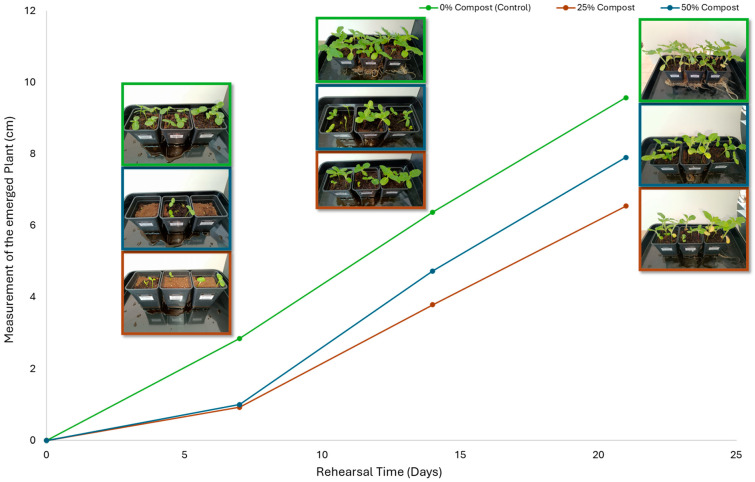
Sunflower growth evolution during the plant growth test.

**Figure 9:**
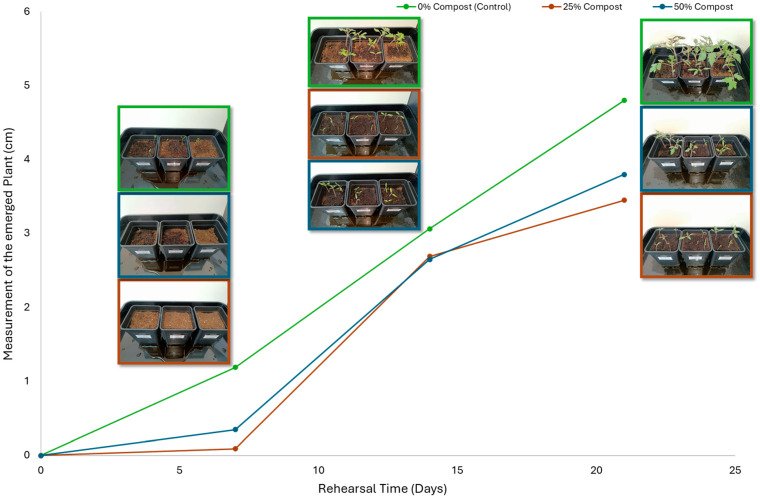
Tomato growth evolution during the plant growth test.

**Figure 10:**
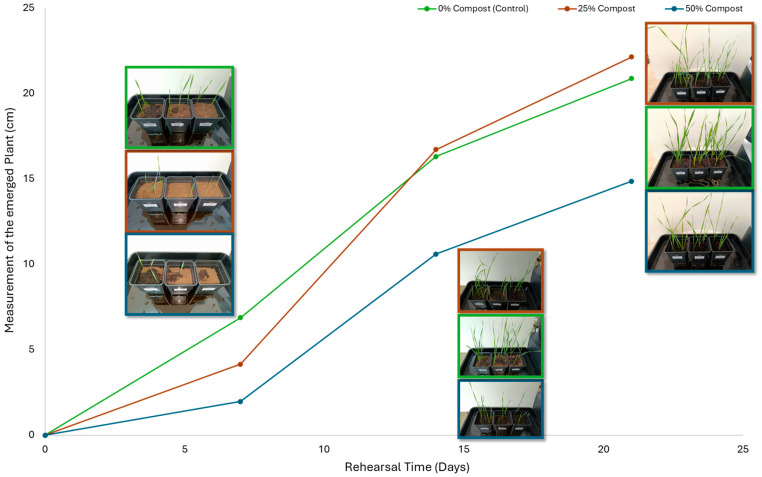
Wheat growth evolution during the plant growth test.

Subsequently, a second assay was conducted to evaluate the phytotoxic effects on plant growth. The growth progression of the selected plant species barley (*Hordeum vulgare*), onion (*Allium cepa*), sunflower (*Helianthus annuus*), tomato (*Solanum lycopersicum*), and wheat (*Triticum aestivum*) throughout the 21-day experimental period is illustrated in Figure 6, Figure 7, Figure 8, Figure 9 and Figure 10.

The growth rate (*GR*) for each plant was determined by Equation (5):

*GR*(%) = [PL*_T_*(cm)/*PL_C_*(cm)] × 100, (5)

where PL_*T*_ is the arithmetic average of the plant length from the test triplicate in centimeters, and PL_C_ is the arithmetic average of the plant length from the control in centimeters. On the other hand, the survival rate (*SR*) was determined using Equation (6):

*SR*(%) = [(N_PE_ − N_PW_)/N_PE_] × 100, (6)

where for each species, N_PE_ is the number of plants that emerged and N_PW_ is the number of plants that withered. 

To assess the phytotoxic effects on leaf morphology across the five plant species examined in this study, the corresponding results are compiled and presented in Table 6.

**Figure 11:**
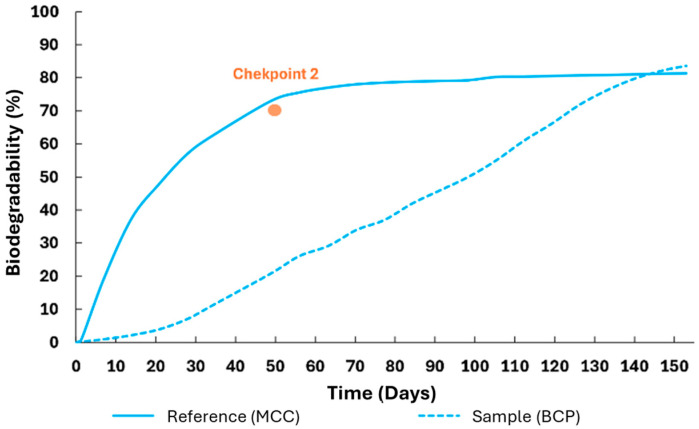
Sample (BCP) and Reference (MCC) biodegradability test results.

**Figure 12:**
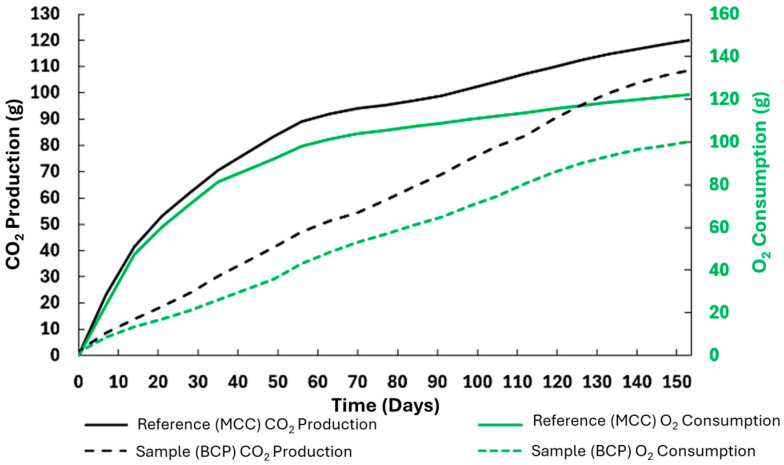
Evolution of O_2_ consumption and CO_2_ production throughout the biodegradability test.

Regarding the test of biodegradation under thermophilic conditions (58 °C) [15], the TOC of both the MCC reference and the BPC sample was determined, with results of 43.3% and 48.2%, respectively. This test was carried out, burying the samples under appropriate soil, for 155 days, reaching 81% biodegradability, as shown in Figure 11. Additionally, Figure 12 shows the evolution of O_2_ consumption and CO_2_ production throughout the experiment.

All the aforementioned biodegradability test checkpoints were attained. In addition, at the end of the test (day 155), all reaction vessels were searched for any visible remaining pieces of the samples, and none were found. Furthermore, in the final stage of the test, the samples’ biodegradability had reached a plateau phase (Figure 11), confirming the validity and success of the experiment.

## 4. Discussion

Figure 1 depicts a FTIR-ATR spectrum composition comparing the 3 samples under study. The spectra show typical PLA bands for all samples, such as the intense peak characteristic of the carbonyl group (C=O) of PLA at 1750 cm^−1^, and the typical C–H stretching bands in the range 3000–2800 cm^−1^. As expected, an additional broad band associated with –OH groups between 3500 and 3000 cm^−1^ can be spotted, which derives the cellulose fibers present in the materials. In all three spectra, this band has moderate intensity, indicating low humidity or good fiber dispersion. Between 1500 and 900 cm^−1^ a complex region with C–O and C–C bands, which can be associated with both PLA and cellulose, can also be identified. Out of the three spectra, the BCP spectrum shows the highest band definition, suggesting the material endured less thermal processing than the others. Samples IP1 and IP2 show similar spectra, with just small variations in band intensity, particularly in the range 1000–1200 cm^−1^ (C–O) that are probably derived from differences in molecular orientation or thermal degradation during the injection process. Noteworthily, neither of the IP1 and IP2 spectra exhibit new bands relative to the BCP spectrum, implying the absence of meaningful chemical reactions during processing. Thus, FTIR analysis confirms that the chemical structure of PLA and cellulose was preserved during the injection process, since the only observable differences can be attributed to physical effects (orientation, crystallinity) and not to chemical alterations, therefore confirming the “fingerprint” of this biocomposite. Previous work has shown similar behavior in PLA–cellulose biocomposites, also portraying practically unchanged spectra after processing. Differences in band intensity can be attributed to structural reorganization and decrease in PLA crystallinity [34,35].

Based on Table 3, we can see that the injection molding process of the BCP itself was majorly responsible for a reasonable increase in the CrI of the molded structures from 15.8% in the case of the BCP to 52.4% and 49.4% for IP1 and IP2, respectively, as a result of favored chain orientation and PLA recrystallization. The potentially different operating conditions resulting from the injection process of each of the parts (such as differences in the flow rate of the material and the cooling speed of the structures) did not contribute to substantially affecting the crystallinity of the final structures. In this sense, the rapid composting of the BCP can be attributed not only to the large specific surface area but also to factors such as the greater quantity of amorphous regions of the biocomposite, while the increased crystalline denser regions of IP1 and IP2 hinder the penetration of water and enzymes, delaying hydrolysis and enzymatic digestion of PLA. Studies demonstrate that injected PLA samples exhibit a lower disintegration rate (<60% in 12 weeks) compared to 3D printed versions (<90% in the same period) [36]. On the other hand, highly crystalline materials tend to persist longer in the environment, increasing the risk of microplastic formation. These remain biologically active for longer, although PLA does not contain intrinsically toxic substances [37]. Present cellulosic fibers, despite contributing to water transport into the structure and also serving as a substrate for microorganisms, concomitantly act as nucleating agents, increasing the crystallinity of PLA. However, the dominant effect is to delay overall degradation due to the structural barrier [38]. Thus, differences observed in the disintegration time during the composting of IP1 and IP2 are due exclusively to the different specific surface area of the samples and, to a lesser extent, to the possible disparity in the distribution of cellulose fibers between these two samples.

During the compostability test conducted under thermophilic incubation, the material exhibited darkening, absence of odor, and an earthy appearance, alongside complete disintegration by day 45 (Figure 3), confirming the maturity of the compost and the effectiveness of the composting process. In addition to achieving 100% disintegration across all three reactors for the 3 samples (BCP, IP1, and IP2), the near-neutral pH (pH between 6 and 8) of the final compost and the reduction in volatile solids content relative to that of the initial synthetic solid waste further validated the reliability of the composting assay (see Table 4). Smaller initial sample dimensions resulted in a less pronounced reduction in volatile solids content at the end of the composting process compared to the initial values.

According to the compostability results, the time required for complete disintegration of the material during composting is closely related to its exposed surface area, since the IP1 pieces (with a higher specific surface area) disintegrated in less time than the IP2 ones and this effect is ever more pronounced for the BCP sample (Figure 5). This agrees with several reports made by other authors [39,40,41]. Furthermore, the disintegration rate seems to be more pronounced at an early stage and slows down over the incubation period (Figure 4). This may also be related to the composition of the studied material: the literature reports that, although the cellulose present in a polymeric matrix favors the transport of water into the structure, accelerating the initial hydrolysis and its swelling, the amorphous parts of the material are firstly degraded, translating into an increase in the materials’ degree of residual crystallinity over time, and on the other hand decreasing the degradation rate at a later stage of the process. Thus, the relationship between the size of the pieces remaining throughout the test and its duration is not directly proportional [42,43]. In addition, the distribution of cellulose fibers within the polymer matrix may not be uniform, since in the case of injection molded parts, there may be accumulation at the base due to gravity while the material is hot enough for element mobility in the bulk, or due to the preferential orientation induced by the flow that occurs during the injection molding process [44,45]. This non-uniform dispersion of fibers in the polymer matrix certainly influences the total available surface area, further emphasizing the role of this parameter in compostability.

Likewise, in the biodegradability assay of the BCP sample, it is possible to infer from Figure 11 that, up to approximately 25 days of incubation, there was an early phase of very slow degradation, which probably corresponds to the transport of water into the structure and swelling. This was followed by a more accelerated degradation until approximately day 140 and again in the end a slower phase until a plateau was reached. This profile certainly corresponds not only to a structural change in the material but also to the aforementioned increase in the materials’ residual crystallinity. Concordant results were reported by Kulikowska et al. (2020) [46]. When the biodegradation profile of the BCP sample is compared to that of the reference material, it is clear that there is no initial phase of slow degradation for the reference and instead it ramps up exponentially from day 1. This is in line with the expectations, since the MCC used as a reference, despite not containing amorphous material, has a much higher specific surface area compared to the BCP sample (microcrystalline cellulose had a particle size of only 20 µm), so the initial degradation is accelerated, reaching the plateau phase around day 60. Noteworthily, despite the extensive biodegradability test, neither the BCP sample nor the reference material achieved 100% biodegradation (although the compostability test achieved 100% observable disintegration, this does not mean that complete biodegradation occurred). The final calculated biodegradability of the sample (81%) was higher than that of the reference material (79%). Although the calculated biodegradability could be slightly increased had the assay been prolonged, a major upsurge was not expected, since both materials had seemingly attained a plateau phase by the time the test ended on day 155. The registered incomplete biodegradation, although seemingly conflicting, is actually expected: authors such as Fritz et al. (2025) [47] reported that biodegradation methods based on the quantification of CO_2_ released, such as the undertaken respirometric method, underestimate the true final biodegradability of the materials, since part of the elemental carbon (estimated through the TOC) is transformed into microbial biomass (cellular protein). Thus, it is logical to infer that an accelerated initial biodegradation rate may also correspond to a superior production of microbial biomass, lowering the final biodegradability calculated through the evolved CO_2_, which is a limitation of the method.

The results presented in Figure 12 from respirometric analysis over 150 days reveal some differences between the MCC (reference) and BCP (sample) groups, both in CO_2_ production and O_2_ consumption. The MCC group consistently showed higher values in both metrics, suggesting greater microbial activity or metabolic efficiency in degrading the tested substrate. In the MCC group, cumulative CO_2_ production reached approximately 120 g, while the BCP group attained approximately 90 g. Following the same trend, O_2_ consumption was approximately 140 g and 100 g for MCC and BCP, respectively. Thus, the gas consumption and exhaustion profiles indicate that MCC promoted a more intense microbial respiration, possibly due to a greater availability of biodegradable carbon and/or greater microbial activity. Kintzi et al. (2025) [48] reported similar CO_2_ production curves in respirometric systems applied to the determination of polyethylene glycerol biodegradation by activated sludge microbiomes, to which the registered discrepancy was attributed precisely to the substrate type and microbiological composition. Zhang et al. (2024) [49] pointed out that the calculation of gas consumption and exhaustion during microbial respiration can be estimated in systems where nitrification occurs, reinforcing the importance of considering the origin of gas consumption in data interpretation. Furthermore, Vaziourakis et al. (2025) [50] demonstrated that the Respiratory Quotient (RQ), a concept they defined as the molar ratio between CO_2_ produced and O_2_ consumed in biodegradation processes, varies noticeably between ecosystems and is largely correlated with not only the energy availability of the organic matter undergoing degradation but also with the metabolic profile of the microbiome present in the system. In the current study, the RQ estimated for the MCC is close to 0.86, while for the BCP it is slightly higher (0.90), translating into a predominantly aerobic microbial respiration, with possible variation in the conversion efficiency of the organic matter present in the sample due to the composition of the substrate, the existing microbial population, or, eventually, the potential presence of metabolic inhibitors hindering their activity. Thus, the lower activity detected in the BCP group may hypothetically be related to lower carbon bioavailability or a microbial population less adapted to the sample. According to reports from other authors, the advantages of incorporating cellulose into PLA are evident: in thermophilic environment composting (58–60 °C), pure PLA exhibits slow degradation, with mass loss of less than 1% in the first 30 days and significant mineralization only after 90 to 180 days [46,51]. Prior to the microbial attack on the material, hydrolysis of the polymeric chains and consequent reduction in molecular weight needs to take place, hence the initial low degradation rate [52]. Nonetheless, PLA biocomposites with incorporated cellulose fibers (micro or nanocellulose) actually accelerate the initial degradation process, since the fibers increase water absorption and create points of weakness in the matrix, allowing for greater microbial colonization [24,52]. These results agree with the results obtained in the present work, since the BCP sample completely disintegrated after a relatively short amount of time under thermophilic composting conditions (45 days). Moreover, studies found in the literature indicate that, under controlled conditions, PLA-nanocellulose composites can achieve biodegradation rates up to 10–15 times higher than those of pure PLA, with complete mineralization in about 90 days [24,52]. In our study the BCP sample had micronized cellulose incorporated in the polymeric matrix instead of nano-sized cellulose and attained a maximum biodegradation (reaching a plateau phase) at 155 days of thermophilic respirometric biodegradation, which is in line with what was described by Macedo et al. (2017), indicating that microcellulose also accelerates biodegradation, but less intensely than nanocellulose [24].

Regarding the ecotoxicity tests carried out with the mature compost from BCP composting, the results revealed distinct effects depending on the species tested, both in seed germination tests and in plant growth tests. On the one hand, the germination tests demonstrated that the compost had no substantial effects on barley seeds, while contrarily having a strong fertilizing effect on wheat, evidenced by a GI of 174.2%. As for sunflower seeds, a GI of 77.8% denoted a moderate phytotoxic effect (Table 5), revealing the negative impact that the mature compost had on initial root growth. Conversely, plant growth tests revealed a moderate phytotoxic effect for barley and wheat (Table 6). This adverse effect may be associated with the presence or accumulation of residual harmful compounds and/or the dynamics of nutrient release. According to studies by other authors, composts derived from biopolymers can exhibit specific features, such as gradual release of nutrients, presence of oligomers resulting from the biopolymer chain degradation, and residual organic acids, in addition to changes in the C/N ratio and salinity of the substrate [53]. All these characteristics can affect plant species in different ways. In this work, barley, sunflower, and tomato (Figure 6, Figure 8 and Figure 9) recorded the highest growth rate in the control group, while the addition of compost, mostly at 50%, substantially reduced the growth rate. This behavior may be related to the aforementioned release of soluble salts or organic acids, combined with the alteration of the C/N ratio, at levels that caused osmotic stress, limiting the absorption of water and nutrients by the plants. The high microbial activity stimulated by compost may also have competed for nitrogen, reducing its availability [54]. In the onion plants (Figure 7), the opposite effect was observed: the highest growth rate occurred with 50% compost, suggesting that this species might tolerate high concentrations of organic matter and benefit from higher water retention and the gradual release of nutrients from the degradation of PLA and cellulose fibers [54]. Furthermore, since onions have a shallow root system, the improvement in the physical structure of the substrate and the availability of nutrients may have favored the plant’s growth. The case of wheat (Figure 10) is particular: 25% compost increased the growth rate, while 50% reduced this parameter. This behavior can be explained by the balance between benefits and adverse effects: while in moderate proportions (25%) the compost can have a fertilizing effect, when increasing the availability of nutrients and favoring the physical structure of the soil, on the other hand at higher proportions (50%), the negative effects prevail over the benefits affecting the physiological processes of the plants [53,55]. In general, these results indicate that the influence of compost on the growth rate of plants varies between species and is related to its chemical composition and degree of maturity. In moderate concentrations, the compost can act as a soil conditioner, improving nutrient availability and water retention; at high concentrations, however, the risks of salinity, nutritional imbalance, osmotic stress, and root hypoxia increase, reducing the plant’s growth rate. These findings reinforce the importance of adjusting the compost dose according to the species and ensuring its complete stabilization before its application in the soil, particularly when the compost is derived from biopolymers such as PLA, which can generate byproducts during degradation with possible phytotoxic effects [56]. These results are corroborated by previous studies, such as those by Lončarić et al. (2024) [57], which emphasize the relevance of specificity associated with plant species variation in assessing compost maturity and its safety when applied in the environment.

The species-dependent effects observed in seed germination and plant growth assays can also partially be explained by the chemistry of residual compounds involved in the biocomposite degradation process: PLA degradation products, such as lactic acid, might be responsible for a slight decrease in the pH of compost obtained from BCP composting, as it exhibits a pH level very close to neutral but still slightly acidic (Table 4), possibly inhibiting the development of physiologically acid-sensitive species, while sugars and oligosaccharides from the degradation of cellulose can act as a carbon source, stimulating the growth of other species. Furthermore, secondary metabolites derived from lignin degradation, such as traces of phenolic compounds and aromatic aldehydes, might exert allelopathic effects, selectively modulating the germination and plant growth process, as higher temperatures applied in thermophilic composting favor the release of these metabolites, which can persist in the final compost [52,58].

The results obtained thus reinforce the need for complementary phytotoxicity tests covering a vast and differentiated number of plant species at different stages of their life cycle for a more robust and discerned ecotoxicological characterization, since the absence of harmful effects resulting from contact with certain composts during the seed germination phase, for example, does not guarantee by itself a healthy growth free from phytotoxic effects at a later stage of plant growth, and vice versa.

## 5. Conclusions

Based on the results presented, this study confirmed the efficient composting and biodegradation of the biocomposites tested under controlled thermophilic incubation. Complete disintegration of all samples (BCP, IP1, and IP2) was achieved during the composting processes, and the maturity of the resulting composts was verified by visual, olfactory, and chemical indicators, such as darkening, an earthy texture, absence of odor, a pH close to neutrality, and a reduction in volatile solids content compared to the initial SSW.

The disintegration rate of the samples was largely influenced by the exposed surface area, with pieces with a higher specific surface area disintegrating at a faster rate. This result was particularly evident in the BCP sample and is consistent with results reported in the literature. The differences in the degradation dynamics between samples were also likely a consequence of the uneven distribution of cellulose fibers within the polymer matrix, especially in the injection-molded pieces. The biodegradability analysis revealed a multiphase degradation profile for the BCP sample, with an initial slow phase followed by an accelerated but steady degradation and a final plateau phase. In contrast, the reference material, MCC, underwent accelerated degradation from the outset, largely due to its greater specific surface area. Although the biodegradability determination tests met the checkpoints that attest to their validity, none of the materials tested achieved 100% calculated biodegradability. Furthermore, and contrary to what might be expected, the BCP sample attained a slightly higher final biodegradability than that of the MCC (83% versus 79%, respectively). This discrepancy is likely due to limitations inherent to the methodology, given that determining biodegradability by respirometric methods, that is, based on the determination of evolved CO_2_, entails the possibility of underestimating the true final biodegradability by not accounting for the carbon integrated into the microbial biomass during population growth. Indeed, the results from the evolution of consumption and exhaustion gases throughout the tests were in accordance with a greater microbial activity in the MCC, with higher registered cumulative CO_2_ production and O_2_ consumption. The estimated RQ values were consistent with a predominantly aerobic microbial respiration, with small differences in substrate conversion efficiency between the materials tested.

Ecotoxicity tests revealed responses to the presence of the mature compost strongly dependent on the plant species and the plant’s life cycle phase. Although wheat experienced a fertilizing effect and barley was not meaningfully affected during the germination phase, both species exhibited markedly phytotoxic effects during later stages of plant growth, likely due to the presence or accumulation of residual compounds detrimental to plant metabolic processes or nutrient release dynamics. In contrast, onion, sunflower, and tomato showed no signs of phytotoxicity, indicating greater tolerance or adaptation.

Overall, the results confirm the compostability and biodegradability of the tested materials while emphasizing the influence of physical structure, fiber distribution, and methodological limitations on degradation performance. Furthermore, the ecotoxicological outcomes highlight the need for phytotoxicity assessments targeting plant species, plant development stages, and the added compost quantity to ensure safe agricultural application of composted products.

## Figures and Tables

**Table 1:** Volatile solids content results obtained for the 3 samples.

Sample		Volatile Solids Content (%)
Biocomposite Pellets (BCP)	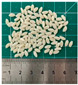	99.9 ± 0.03
Injected Part 1 (IP1)	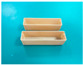	99.9 ± 0.04
Injected Part 2 (IP2)	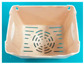	99.2 ± 0.17

**Table 2:** Results obtained in the determination of heavy metals and other toxic substances for the samples under study.

Element	Fluoride (F)	Arsenic (As)	Cadmium (Cd)	Lead (Pb)	Cobalt (Co)	Copper (Cu)	Chromium (Cr)	Mercury (Hg)	Molybdenum (Mo)	Nickel (Ni)	Selenium (Se)	Zinc (Zn)
European Limit (mg/kg)	100	5	0.5	50	N.A. ^1^	50	50	0.5	1	20	0.75	150
BCP (mg/kg)	<20	<1.3	<0.1	<0.1	<2.5	<2.5	<2.5	<0.1	<0.1	<2.5	<0.5	<2.5
IP1 (mg/kg)	<20	<0.5	<0.25	<0.5	<0.25	0.23	<0.25	<0.1	<0.25	<0.25	<0.5	<2.5
IP2 (mg/kg)	<20	<0.5	<0.25	<0.5	<0.5	0.23	<0.25	<0.1	<0.25	<0.25	<0.5	<0.5

^1^ N.A.—Not Applicable (no document, to our knowledge, imposes a limit on this element).

**Table 3:** Calculated results for the CrI by the Segal method for the three samples.

Reactor	BCP	IP1	IP2
I_002_ (crystalline peak, 2θ ≈ 21–23°)	1326	3098	3078
I_am_ (amorphous region, 2θ ≈ 17–19°)	1117	1475	1557
Crystallinity Index (CrI) (%)	15.8	52.4	49.4

**Table 4:** Average obtained results for the disintegration test for the three samples.

Reactor	BCP	IP1	IP2
Odor	Odorless	Odorless	Odorless
Visual Appearance	Earth-like dark color	Earth-like dark color	Earth-like dark color
Time (days)	45	106	141
pH	6.85 ± 0.10	7.78 ± 0.08	7.29 ± 0.08
SSW Volatile Solids Content (%)	76.4 ± 1.7	78.2 ± 1.4	77.2 ± 1.4
Mature compost Volatile Solids Content (%)	73.3 ± 0.8	66.2 ± 2.5	62.7 ± 0.4
C/N ratio	36.6 ± 0.4	33.1 ± 1.2	31.3 ± 0.2
Dry Mass of Residual Sample (g)	0	0	0
Degree of Disintegration (%)	100	100	100

**Table 5:** BCP compost evaluation results obtained for the germination seed tests.

Germination Seed Test	Image at 72 h	Germination Index (%)	Evaluation
Barley	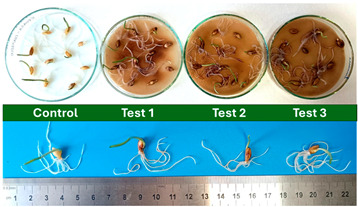	96.2	80 < *GI* < 100 → Not phytotoxic mature compost
Wheat	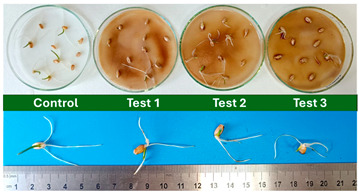	174.2	*GI* > 100 → The compost enhances the germination and growth of the plant’s roots (strong fertilizer effect)
Sunflower	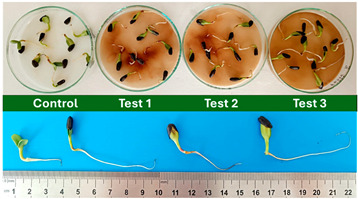	77.8	60 < *GI* < 80 → Moderately phytotoxic mature compost

**Table 6:** Plant growth tests results of phytotoxic effects on the leaf’s morphology.

Growth Plant Test	Compost	Growth Rate (%)	Survival Rate (%)	Photo 1st True Leaves	Phytotoxic Effects
Barley	0% (Control)	100	100	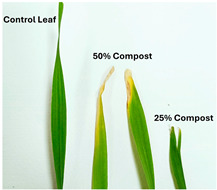	▪ Necrosis and leaf margin burn ▪ Chlorosis ▪ Leaf deformation: Fissures
	25%	74.5			
	50%	78.0			
Onion	0% (Control)	100	100	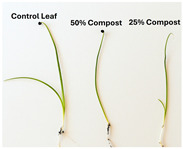	▪ Leaves without phytotoxic effects
	25%	92.2			
	50%	89.4			
Sunflower	0% (Control)	100	100	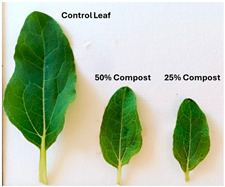	▪ Smaller leaves without other phytotoxic effects
	25%	78.9			
	50%	82.6			
Tomato	0% (Control)	100	100	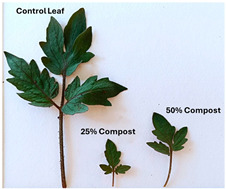	▪ Smaller leaves without other phytotoxic effects
	25%	60.3			
	50%	72.0			
Wheat	0% (Control)	100	100	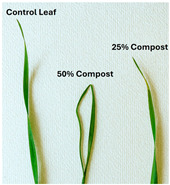	▪ Leaf deformation ▪ Necrosis
	25%	86.8			
	50%	74.0			

## Data Availability

The original contributions presented in this study are included in the article.

## Abbreviations

The following abbreviations are used in this manuscript:

PLA	Polylactic Acid
PS	Polystyrene
PET	Polyethylene Terephthalate
MFC	Micro-fibrillated cellulose
BEKP	Eucalyptus kraft pulp
MFI	Melt flow index
SSW	Synthetic Solid Waste
CO_2_	Carbon Dioxide
EN	European Norm
ISO	International Standard Organization
OECD	Organization for Economic Cooperation and Development
SEM	Scanning Electron Microscopy
FTIR-ATR	Attenuated Total Reflection Fourier Transform Infrared Spectroscopy
X-RD	X-ray Diffraction
CrI	Crystallinity Index
I_002_	Maximum intensity of the crystalline peak
I_am_	Minimum intensity in the amorphous region
BCP	Biocomposite Pellets Sample
IP1	Injected Part 1 Sample
IP2	Injected Part 2 Sample
ICP-AES	Inductively Coupled Plasma Atomic Emission Spectroscopy
F	Fluoride
As	Arsenic
Co	Cobalt
Cr	Chromium
Cu	Copper
Mo	Molybdenum
Ni	Nickel
Se	Selenium
Zn	Zinc
Cd	Cadmium
Hg	Mercury
Pb	Lead
TOC	Total Organic Carbon
MCC	Microcrystalline Cellulose Reference
C/N ratio	Carbon/Nitrogen ratio
GI	Germination Index
RG	Relative Germination
RRL	Relative Root Length
N_GS,*T*_	Arithmetic average of the number of germinated seeds from the test triplicate
N_GS,C_	Arithmetic average of the number of germinated seeds from the control
L_R,*T*_	Mean root length of the test triplicate
L_R,C_	Mean root length of the control
GR	Growth Rate
PL_*T*_	Arithmetic average of the plant length from the test triplicate
PL_C_	Arithmetic average of the plant length from the control
SR	Survival Rate
N_PE_	Number of Plants that Emerged
N_PW_	Number of Plants that Withered
O_2_	Oxygen
